# Assessing the impact and implications of the revised Act on the Aggravated Punishment of Specific Crimes in preventing child traffic injuries in school zones in Korea: an interrupted time series analysis

**DOI:** 10.4178/epih.e2024032

**Published:** 2024-02-21

**Authors:** Hong Jin Ku, Jin-Hwan Kim, Young June Choe, Seung Ah Choe, Mark R. Zonfrillo

**Affiliations:** 1 Korea University College of Medicine, Seoul, Korea; 2 Institute of Health and Environment, Seoul National University, Seoul, Korea; 3 Department of Pediatrics, Korea University Anam Hospital, Seoul, Korea; 4 Departments of Emergency Medicine and Pediatrics, Alpert Medical School of Brown University, Providence, RI, USA

**Keywords:** Pediatrics, Child, Traffic, Accidents, Injury

## Abstract

In 2019, a child’s death in Korea led to legislation that imposed stricter penalties for school zone traffic violations. We assessed the impact of that legislation using 2017-2022 Traffic Accident Analysis System data. Adjusted analyses revealed a significant decline in severe injuries in school zones, decreasing from 11 cases to 8 cases per month (p=0.017). The legislation correlated with a reduced risk of all child traffic injuries (risk ratio, 0.987; 95% confidence interval, 0.977 to 0.997; p=0.002), indicating its efficacy in curbing accidents.

## GRAPHICAL ABSTRACT

[Fig f2-epih-46-e2024032]

## Key Message

Revised legislation in Korea, following a child’s tragic death, enforced stricter penalties for school zone traffic violations. Analysis of 2017-2022 data showed a significant decrease in severe injuries from 11 to 8 cases monthly (p=0.017). The legislation also reduced the risk of all child traffic injuries (risk ratio, 0.987; 95% CI, 0.977 to 0.997; p=0.002), demonstrating its effectiveness in preventing accidents. This study underscores the legislation’s positive impact on child safety in school zones, emphasizing the importance of ongoing enforcement efforts for road safety.

## INTRODUCTION

Traffic injuries remain a predominant cause of child and adolescent fatalities worldwide, calling for a comprehensive understanding of their varied dynamics and outcomes [1]. Previous studies have underscored the significance of this issue and highlighted the marked disparities in road injury patterns based on geographic location [2,3].

Pediatric injuries from road traffic accidents are a notable healthcare challenge in Korea that extends beyond the confines of medical facilities. The concept of school zones, as defined by Korea’s Road Traffic Act (Article 12), empowers local authorities to enforce a maximum speed limit of 30 km/hr in school zones to enhance child safety [4]. However, a tragic incident in 2019, involving the unfortunate demise of a 9-year-old boy within a school zone, led to revisions of the Road Traffic Act and the Act on the Aggravated Punishment of Specific Crimes, imposing heavier punishments for traffic accidents in school zones.

However, the implementation of heavier punishments has not been without controversy. Some critics have asserted that the punitive measures are excessive and misaligned with the principles of criminal law. Concerns have also been raised regarding the potential unintended consequences of setting speed limits too low, because some studies have suggested that extremely restrictive speed limits are less effective in reducing speeds [5].

This study aimed to assess the efficacy of the new heavier punishments in preventing child traffic injuries and death. This study adopted a comparative approach to assess the law’s impact, with the goal of enhancing child safety within school zones.

## MATERIALS AND METHODS

### Study population and setting

This retrospective observational study encompassed all traffic injuries and deaths that occurred between January 2017 and December 2022 in Korea. As of 2023, the estimated population of Korea was 51 million, with approximately 5.9 million children enrolled in K-12 schools [6]. The majority of public and private schools in Korea follow standardized semester schedules, although minor variations in dates exist among individual institutions and regions. Typically, the academic year commences in early March, with the first semester extending until late July. Following a summer recess, the second semester generally runs from mid- to late August through mid-February, including a winter break from January to mid-February.

A school zone refers to a designated area around a kindergarten or school, recognized as necessary for protecting children from the risks of traffic injury [4]. Within school zones, the speed limit for vehicles, including cars and surface trams, should be restricted to 30 km/hr (per Article 12, paragraph 1 of the Road Traffic Act and Article 14 of the Enforcement Decree of the Road Traffic Act).

As of August 2022, there were 11,710 elementary, middle, and high schools across the country, with approximately 16,000 designated school zones near these schools.

### Data

We obtained official traffic injury and death data for the years 2017 to 2022, as registered by the National Police Agency of Korea (https://taas.koroad.or.kr/). This comprehensive dataset was sourced from the Traffic Accident Analysis System (TAAS) and included police-reported road traffic incidents. While the dataset may not include all minor incidents that go unreported to the police, it remains a valuable resource for estimating the prevalence of incidents, injuries, and fatalities. For this study, we included pedestrian-to-motor (i.e., pedestrian struck) injuries occurring in children aged ≤ 12 years in school zones. The outcome measures were all reported pedestrian-to-motor injuries in school zones, including mild injuries (not requiring hospitalization), severe injuries (requiring hospitalization), and deaths occurring within 30 days of a traffic accident.

### Intervention

The revised Act on the Aggravated Punishment of Specific Crimes was first initiated on October 15, 2019, by the National Assembly and was enacted on March 25, 2020. The fundamental premise of the law revolves around imposing more substantial penalties on drivers found responsible for accidents within school zones. Central to the revised law is the provision stipulating that drivers causing the demise of a child aged ≤ 12 years due to a traffic violation within a school zone can be subjected to a mandatory minimum term of 3 years, up to life imprisonment. When the transgression leads to injury, the offender potentially faces imprisonment for a minimum of 1 year, up to a maximum of 15 years. The alternative punitive option involves a monetary fine of up to 30 million Korean won (approximately US$22,000). Legislative notices of upcoming laws are collectively announced prior to their enactment, in January of the corresponding year. Therefore, we defined 2 periods as follows: January 2017 to December 2019 (36 months) was considered the before period, and January 2020 to December 2022 (36 months) was the after period.

### Model

Our model analyzed all police reports of traffic incidents within school zones that resulted in bodily injury or death. Death was defined as mortality within 30 days of the traffic accident. In our model, we incorporated the month as a continuous variable, while also introducing the categorial variables of season (spring, summer, fall, and winter) and the number of days school was open each month. We scrutinized the frequencies of traffic injury and death both before and after the enhanced law intervention. Potential heterogeneity was evaluated using the Wilcoxon rank sum test. We also conducted 3 robust interrupted time series analyses utilizing Poisson regression models, as detailed in the provided reference [7] and shown below:


(1) $$\mathrm{Interventional}\;\mathrm{effect}\;\mathrm{only}\;\mathrm{model}:\;\log\left({\text{Y}}_{t}\right)=\beta_{0}+\beta_{1}T+\epsilon_{t}$$

Segmented regression model considering the linear effect of time (month), seasonality, and open school days per month:


(2) $$\log\left(Y_{t}\right)=\beta_{0}+\beta_{1}T+\beta_{2}X_{t}+\beta_{3}P_{t}+f\left(t\right)+\epsilon_{t}$$

Adjusted model for trend and common confounders using a control series:


(3) $$\log\left(Y_{t}\right)=\beta_{0}+\beta_{1}T+\beta_{2}X_{t}+\beta_{3}G+\beta_{4}P_{t}+\beta_{5}\mathrm{GT}+\beta_{6}{\mathrm{GX}}_{t}+\beta_{7}{\mathrm{GP}}_{t}+f\left(t\right)+\epsilon_{t}$$

In these formulae, *Yt* is the expected frequency at the time point T=t. *Xt* represents the presence (after the intervention) or absence (before the intervention) of the intervention. G in the control series model denotes the group to which observations belong (intervention group and control group), and *P_t_* means the time (months) after the intervention. *β*_0_ is the baseline incidence before the intervention, *β*_2_ is the effect of the intervention, *β*_3_ is the baseline group difference, *β*_4_ is the slope after intervention, *β*_5_ is the group difference in slope before the intervention, *β*_6_ is the after-intervention group difference (level impact), *β*_7_ is the group difference in slope before the intervention (slope impact), *ϵ_t_* is an error at time *t* that may show autocorrelation. The combined effect of the potential confounders including month, seasonality, and open school days in each month is presented by *f(t)*.

We calculated the adjusted risk ratio (RR) and 95% confidence interval (CI) of all traffic injuries after the new law was introduced. Analyses were performed using R version 4.3.3 (R Foundation for Statistical Computing, Vienna, Austria), and statistical tests were 2-tailed at the 5% level.

### Ethics statement

This study was reviewed and approved by the Institutional Review Board (IRB) of Korea University Anam Hospital (IRB No. 2023AN0345). Since this study was a retrospective study, written informed consent was waived by the IRB. The study was conducted in accordance with the provisions of the Declaration of Helsinki and Good Clinical Practice guidelines.

## RESULTS

Between 2017 and 2022, a total of 1,867,183 traffic injuries and 20,047 deaths (1.1% of all injury) occurred in Korea. In children, 40,091 injuries and 116 deaths occurred during the before period, while 32,867 injuries and 65 deaths occurred during the after period.

Table 1 shows baseline features before and after enactment of the stricter school zone traffic law in Korea. The average number of days per month that schools were open and the number of school zones differed between the 2 periods. After adjusting for open school days and number of school zones in the before and after periods, the monthly average of pedestrian-to-motor injuries was 35 (95% CI, 25 to 39) and 29 (95% CI, 23 to 36; p=0.256), respectively; the monthly average number of mild injuries was 20 (95% CI, 13 to 24) and 19 (95% CI, 13 to 26; p=0.919), respectively; while the monthly average number of severe injuries decreased from 11 (95% CI, 8 to 14) to 8 (95% CI, 7 to 11; p=0.017), respectively.

**Figure 1.**
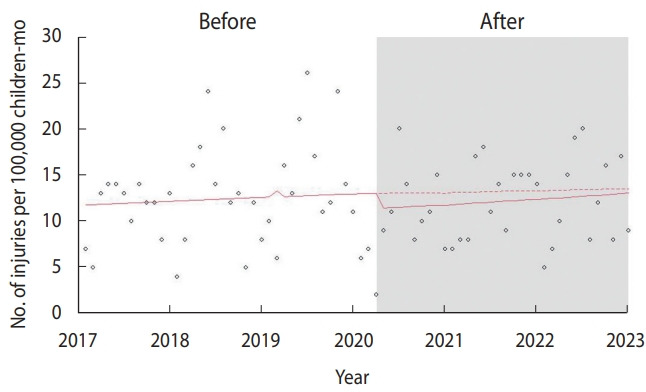
Number of school zone-associated injuries in children (pedestrians) aged ≤12 years in Korea (2017-2022).

The interrupted time series analysis exhibited a pattern of alteration before and after the intervention, with deviation from null effects (Figure 1). All traffic injuries involving children showed an association with the legislative intervention of heavier punishment, indicating a reduced risk of injuries (RR, 0.987; 95% CI, 0.977 to 0.997; p= 0.002).

## DISCUSSION

The present study assessed the effectiveness of the stricter legislation in reducing child traffic injuries, supporting the necessity of an evidence-based approach to shaping legislative and traffic policies. Our findings illustrate the impact of the stricter legislation, revealing a reduction in child traffic injuries after its implementation. Although this outcome recognizes that the enactment of heavier punishment contributed to a decrease in severe child traffic injuries, supplementary measures are warranted to achieve a more substantial reduction in traffic-related incidents affecting children.

In Korea, traffic injuries represent a substantial source of harm to the pediatric population, accounting for 16.7% of injuries among children aged 0-12 years [8]. This is particularly concerning given the upward trajectory of traffic-related incidents in comparison to other sources of harm [9]. Our discussion draws upon insights from existing research and policy paradigms to contextualize these results and suggest avenues for enhancing the effectiveness of heavier punishments. Our study aligns with the perspectives of Bjørnskau & Elvik [10] who asserted that the efficacy of enforcement measures in diminishing road injuries is contingent on their sustained application. Our analysis underscores the importance of consistent enforcement and highlights the potential limitations of relying solely on public-information campaigns, as proposed by Amick & Marshall [11]. The social attention garnered by enactment of the new stricter law is noteworthy; however, the lack of substantial, continued enforcement may hinder the law’s ability to achieve a lasting impact.

Notably, the temporal trends following enforcement of the tougher school zone law revealed a reduction in child traffic injuries. Previous research suggests that while intensified enforcement can yield short-term benefits, sustained outcomes require a more comprehensive approach [12,13]. The World Health Organization emphasizes the importance of evidence-based, stringent measures, supported by continuous enforcement and public education, in reducing road traffic injuries and fatalities [14]. A study by Lee et al. [15] used the Delphi technique and Analytic Hierarchy Process in Korea to prioritize injury prevention and management programs and offers insights relevant to school zone traffic violations. Emphasizing injury surveillance and various prevention strategies, their study aligns with the need for data-driven approaches and comprehensive public health interventions. Their focus on fall prevention, climate change readiness, and prioritized research and development projects reflects an understanding of injury mechanisms, and provides valuable context for evidence-based policies, including those addressing traffic safety in school zones.

There were notable limitations in our study. First, the disruptive effects of the coronavirus disease 2019 (COVID-19) pandemic period (2020-2022) and the unique Korean characteristics, such as extended school days and overlapping commuting hours, warrant cautious interpretation of our findings [6]. Second, it was unclear whether the injuries resulted from pedestrian-versus-motorvehicle accidents or motor-vehicle-versus-motor-vehicle collisions. The enactment of the heavier punishment measures and penalties was largely shaped by public sentiment, rather than an evidencedriven approach. However, the significance of this study lies in its pioneering attempt to evaluate the health implications of legislative and traffic policies using comprehensive national data from the TAAS. Lastly, although we acknowledged the impact of COVID-19 as a potential confounding factor, other policies and interventions aimed at reducing traffic accidents during the study period were not explicitly measured. Therefore, the observed decline in severe injuries and the reduced risk of all child traffic injuries associated with the legislation may be influenced by unmeasured covariates. A more comprehensive understanding of the broader traffic safety landscape and potential confounding variables could enhance the interpretation of the study’s findings.

In conclusion, the enactment of heavier punishment has demonstrated a measure of effectiveness in curbing road traffic injuries in school zones, especially among child pedestrians. However, the reinforcement of penalties alone may be insufficient to achieve substantial reductions in such incidents. To enhance the new law’s efficacy, it is imperative to consider a multifaceted approach that integrates consistent enforcement with targeted education programs for children, fostering heightened awareness of the risks associated with traffic injuries.

## Figures and Tables

**Figure f2-epih-46-e2024032:**
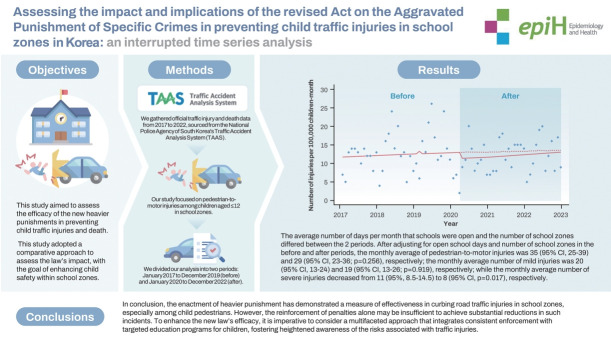


**Table 1.** School zone pedestrian-vehicle injuries in children aged ≤12 years before and after legislation mandating heavier punishment in Korea (2017-2022)

Variables	School zone pedestrian-vehicle injuries in children ≤12 yr		p-value
	Before	After	
Days school was open per month	19 (10-21)	14 (10-20)	0.261
No. of school zones	16,555 (16,355-16,586)	16,896 (16,759-16,912)	<0.001
Monthly average no. of injuries^1^	35 (25-39)	29 (23-36)	0.256
Monthly average no. of mild injuries^1^	20 (13-24)	19 (13-26)	0.919
Monthly average no. of severe injuries^1^	11 (8-14)	8 (7-11)	0.017
Monthly average no. of deaths^1^	0 (0-1)	0 (0-0)	0.271

Values are presented as average (range).

1 After adjusting for the number of days that school was open and the number of school zones per 10,000 school zones (95% confidence interval).
